# Prevention of self-induced re-injury to ruptured globe with a surgically fixated plastic eye shield

**DOI:** 10.1016/j.ajoc.2022.101476

**Published:** 2022-03-09

**Authors:** Gregory Brandon Caudill, Mitchell Jay Wolin, John Delmar Siddens

**Affiliations:** a University of South Carolina School of Medicine Greenville, 607 Grove Rd, Greenville, SC, 29605, USA; b Prisma Health System, Department of Surgery, Division of Ophthalmology and Oculoplastic and Reconstructive Surgery, 104 Simpson St, Greenville, SC, 29605, USA

**Keywords:** Trauma, Open globe, Wound care, Cognitive impairment

## Abstract

**Purpose:**

Open globe injury is an emergent, vision threatening condition. To ensure the best possible visual outcome after an open globe, it is essential to protect the eye from further trauma during the critical period of healing. In cases where the open globe is caused by repeated self-mutilation, long term prevention of re-injury must also be considered and can pose a significant challenge.

**Observations:**

Here we describe a 68-year-old male with a history of severe intellectual disability. The patient presented after an episode of eye self-mutilation, resulting in an open globe injury. After being taken to the operating room for emergent repair of the eye, the primary concern was how to prevent re-injury. Ultimately, as an alternative to the long-term use of restraints, the decision was made to fixate a plastic eye shield over the affected eye using sutures. The eye shield prevented any unwanted manipulation of the eye while implanted, despite several attempts. After 18 days, the eye shield was forcibly removed by the patient. However, this allowed adequate healing time, and there has not been any repeated damage to the eye since.

**Conclusions and Importance:**

Our proposal to suture a plastic eye shield to the orbital rims of a patient is an attempt to allow the eye to heal while avoiding prolonged use of restraints and minimizing long-term hospital stays. This intervention may prove to be beneficial for the population of psychologically or cognitively impaired individuals, as they are often implicated in cases of self-inflicted ocular trauma. To our knowledge, this is the first description of the use of this method.

## 1 Introduction

Open globe injury (OGI) is an emergent, vision threatening condition. Even when patients undergo primary closure of the globe within 24 hours of injury, a no-vision outcome can be expected in approximately 26% of cases, with secondary enucleation being required in up to 20% of cases.^1^ However, with proper treatment and timely repair, many cases (including no light perception presentations of OGI) can see substantial recovery of vision.^1^^,^^2^ In the United States, globe rupture occurs at an average rate of 3.4 per 100,000 individuals. Common causes of globe rupture in adults include workplace injuries, assaults, and motor vehicle accidents.^3^^,^^4^ Self-inflicted, intentional injuries represent only 1% of these cases.^4^ Psychologically or cognitively impaired individuals are often implicated in cases of self-inflicted ocular trauma such as OGI and self-enucleation.^5^, ^6^, ^7^, ^8^, ^9^ Management of self-inflicted, intentional eye injury in a cognitively impaired patient poses a challenge, as these individuals may attempt re-injury if the cognitive deficits that led to the ocular trauma cannot be corrected. Following their initial presentation, these patients are often placed in restraints and/or on 1:1 monitoring.^5^ Prevention of re-injury is pivotal in cases of self-inflicted ocular trauma leading to OGI, as vision recovery is achievable in a significant number of patients.^1^

## 2 Case report

**Fig. 1:**
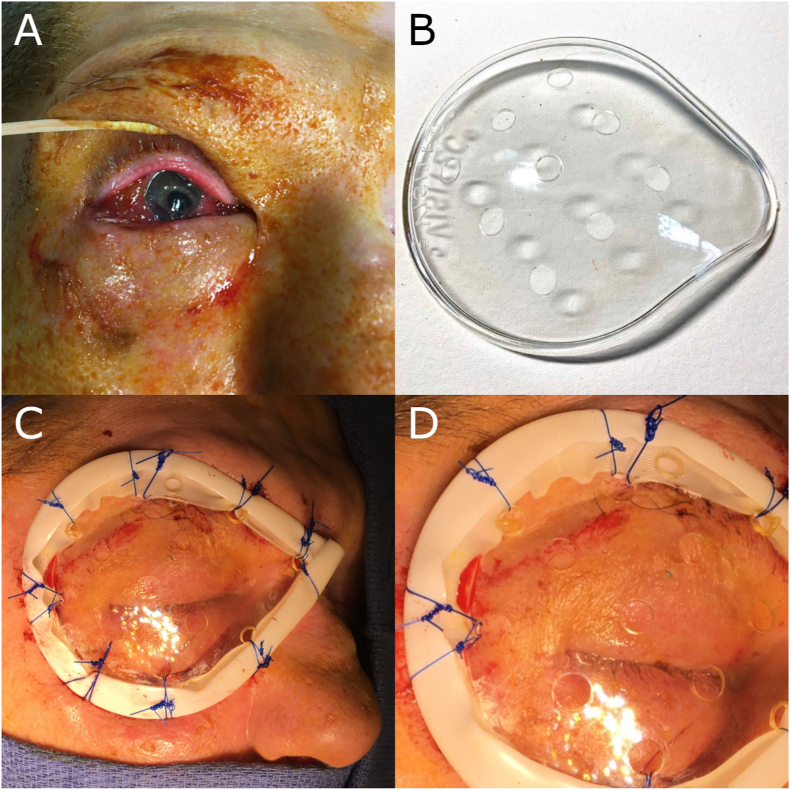
Fig. 1A: Photograph of the open globe injury, prior to repair. Fig. 1B: BVI Visitec, 7.5 × 6.5 cm Universal Eye Shield, prior to modification. Fig. 1C: Photograph of the eye shield after being sutured to the periosteum. Note the1/4 inch Penrose drain that was split in half and fixed to the eye shield for cushioning. Fig. 1D: Close-up photo of the affixed eye shield.

A 68-year-old male with a history of severe intellectual disability presented to the emergency department (ED) via EMS transport from a long-term care home. The ED provider noted a patched right eye exuding a thin, red drainage and surrounded by ecchymosis. After the patch was removed, a pupillary defect was noted. Ophthalmology was consulted, and the patient was taken to the operating room for emergent repair of a suspected ruptured globe (Fig. 1A). During surgery, an inferonasal laceration posterior to the limbus was repaired using five interrupted 8-0 Vicryl sutures. The patient was subsequently admitted with wrist and hand restraints to prevent further harm to the eye. During his hospital stay, repeated attempts to remove his surgical dressing and prod at the recovering right eye necessitated continued use of restraints.

The patient's history indicated that he had been treated several times in the past for minor episodes of self-induced eye trauma. After a visit to the ED two years prior for “poking at his eyes”, ophthalmologists and caregivers noted continued attempts of self-mutilation. Thus, it was deemed unsafe for the patient to have access to his eyes for the duration of his recovery. Although restraints had been used to some success during the patient's hospitalization, regulations in place at his home facility would not allow him to return there while restrained. As an alternative to continued hospitalization for long-term physical restraint, it was decided that surgical fixation of a hard plastic eye shield to the periorbital periosteum of the patient's right orbit would be performed. This is a significant deviation from the routine use of an eye shield, as the shield would normally be fixed over the eye using specialized taping technique. However, it was felt that increased security of the fixated shield would allow the patient to return safely to his home facility.

After obtaining consent from his power of attorney, the patient underwent the procedure as follows: General anesthesia was administered, and the patient's face was prepped with betadine. A modified bvi Visitec, 7.5 × 6.5 cm Universal Eye Shield (Fig. 1B), with additional holes cut for suture placement, was wrapped with a 1/4 inch Penrose drain that was split in half for cushioning. The drain was attached to the eye shield using sutures of 3–0 Prolene. The eye shield was then placed over the right eye and 2–0 Prolene suture was used to secure the eye shield to the periosteal layer of the upper lid, lower lid, and nasal area including the upper nasal canthus. The result was an eye shield that was tightly fixated to the patient's periorbital periosteum (Fig. 1C and D). He was kept inpatient for seven days to observe his behavior on and off restraints. During this time, any eye drops given were administered into the right inner canthus via an angiocath (with the needle removed) inserted through the eye shield.

In the first week following discharge, the patient returned to the ED twice after attempting to remove the eye shield. He was then seen by ophthalmology approximately one week following discharge. The eye shield was noted to be loosened but was still in place at the time. Once again, the benefit of the eye shield preventing further self-inflicted damage to the globe was determined to outweigh any risk due to the inability to access the eye directly. On day 18 following the placement procedure, the patient presented to the emergency room after he successfully removed the eye shield. There was no damage noted to the eye at this time. At this point, it had been approximately four weeks since the globe injury, and the likelihood of the patient attempting to remove the eye shield again was determined to be high. After careful consideration by the ophthalmology team on call, it was determined that the eye shield had remained in place for an adequate period and would not be replaced. The patient was discharged back to his long-term care facility. Subsequent visits with ophthalmology revealed continued healing of the globe without further self-inflicted trauma.

## 3 Discussion

To our knowledge, this is the first time that surgical fixation of a plastic eye shield to prevent re-injury has been described in the literature. Although we felt this intervention was appropriate and justified, we recognize several potential limitations: the invasive nature of the procedure and need for general anesthesia, reduction of access to the eye for the application of drops, and the potential for the patient to tamper with the shield if not supervised. There may also be inherent risks associated with the use of a plastic eye shield in this manner. Potential short-term risks include those associated with general anesthesia as well as risk of injury or infection from the procedure itself. Long-term risks may include peri-orbital skin damage from prolonged pressure from the tightly fixed plastic shield as well as increased risk of amblyopia in those within the amblyopic age group.

The benefit of preventing re-injury to the healing globe during the crucial time of healing may justify the potential negative aspects of this treatment. This is especially true in situations where an alternative intervention is either impossible or impractical. For example, one potential alternative could be daily or twice daily taping of the shield to the patient's face/forehead using benzoin. The purpose of benzoin being increased adherence of the tape. An obvious benefit of taping is that the eye shield can be removed to examine the eye or administer eye drops, however the value of this benefit is dubious in a patient that is difficult to examine at baseline. One major limitation of this solution would be the need for an individual trained in proper taping technique to be present daily. This may not be possible in a community hospital setting where access to ophthalmology-trained staff is limited. Also, a taped shield would be significantly easier for a patient to remove on their own, which may necessitate the use of hand restraints. In this case, the avoidance of hand restraints was an essential part of the justification for suturing the eye shield directly to the periosteum.

## 4 Conclusions

Our proposal of a novel solution to suture a plastic eye shield to the orbital rims of a patient is an attempt to avoid prolonged use of restraints and minimize long-term hospital stays. This is especially important in situations with limited access to ophthalmologic care. The use of a plastic eye shield following open globe injury, either taped or surgically fixated, is not a permanent solution. However, we feel that surgical fixation of the shield as an alternative to taping may provide extended protection while the globe is in a crucial phase of healing. This period of extended protection is especially important for patients at high risk of re-injury, such as those with psychological or cognitive impairment.

## Patient consent

That patient was not consented as this article contains no identifying information and images have been depersonalized.

## Disclosures

No funding or grant support. The following authors have no financial disclosures: GBC, BS; MJW, MD; JDS, DO. All authors attest that they meet current ICMJE criteria for Authorship. No acknowledgements.

## Funding

No funding was received for this work.

## Intellectual property

We confirm that we have given due consideration to the protection of intellectual property associated with this work and that there are no impediments to publication, including the timing of publication, with respect to intellectual property. In so doing we confirm that we have followed the regulations of our institutions concerning intellectual property.

## Research ethics

We further confirm that any aspect of the work covered in this manuscript that has involved human patients has been conducted with the ethical approval of all relevant bodies and that such approvals are acknowledged within the manuscript.

## Authorship

All listed authors meet the ICMJE criteria. We attest that all authors contributed significantly to the creation of this manuscript, each having fulfilled criteria as established by the ICMJE.

We confirm that the manuscript has been read and approved by all named authors.

We confirm that the order of authors listed in the manuscript has been approved by all named authors.

## Contact with the editorial office

This author submitted this manuscript using his/her account in EVISE.

We understand that this Corresponding Author is the sole contact for the Editorial process (including EVISE and direct communications with the office). He/she is responsible for communicating with the other authors about progress, submissions of revisions and final approval of proofs.

We confirm that the email address shown below is accessible by the Corresponding Author, is the address to which Corresponding Author's EVISE account is linked, and has been configured to accept email from the editorial office of American Journal of Ophthalmology Case Reports:

## Declaration of competing interest

No conflict of interest exists.
